# Cardiotoxicity induced by immune checkpoint inhibitor: The complete insight into mechanisms, monitoring, diagnosis, and treatment

**DOI:** 10.3389/fcvm.2022.997660

**Published:** 2022-09-20

**Authors:** Sridha Ganesh, Peng Zhong, Xiaoyang Zhou

**Affiliations:** Department of Cardiology, Renmin Hospital of Wuhan University, Wuhan, China

**Keywords:** immune checkpoint inhibitors (ICIs), immune-related adverse events (irAEs), ICI cardiotoxicity, diagnosis, treatment

## Abstract

Immune checkpoint inhibitors (ICIs) have been taking cancer research by storm as they provide valuable therapeutic benefits to cancer patients in terms of immunotherapy. Melanoma and non-small cell lung cancer (NSCLC) are among the most prevalent cancer varieties that were utilized in ICI trials with many other cancer types being involved too. Despite impressive clinical benefits of overall response rate (ORR), progression-free survival (PFS), etc., ICIs are also accompanied by various immune-related adverse events (irAEs). Amongst the irAEs, cardiotoxicity bags a crucial role. It is of paramount importance that ICI-induced cardiotoxicity should be studied in detail due to its high mortality rate although the prevalence rate is low. Patients with ICI cardiotoxicity can have a greatly enhanced life quality despite adverse reactions from ICI therapy if diagnosed early and treated in time. As such, this review serves to provide a complete insight into the predisposing factors, mechanism, diagnostic methods and treatment plans revolving around ICI-induced cardiotoxicity.

## Introduction

There are various types of ICIs in use to date and they cater to different malignancy categories as depicted in Table 1.

**Table 1:** Common types of ICIs and malignancies they are prescribed for (5–7).

**Type of ICI Inhibitor**	**Example**	**Cancers are mainly used in**
CTLA-4 inhibitor	Ipilimumab, tremelimumab	Melanoma, breast cancer
PD-L1 inhibitor	Avelumab, atezolizumab, durvalumab	Non-small cell lung cancer, breast cancer
PD-1 inhibitor	Pembrolizumab, nivolumab, cemiplimab	Non-small cell lung cancer, melanoma, breast cancer

Cancer cells are notorious for their evasion from immune surveillance by downgrading the role of T-cells in anti-tumor activity and bypassing immune checkpoints. To address the immune surveillance evasion issue, ICI therapy was established to reverse the inhibition of T-cell response to tumor cells and it has been creating a revolution by opening up beneficial survival opportunities for cancer patients. Table 1 listed FDA-approved ICI drugs commonly used for melanoma, breast cancer, and non-small cell lung cancer treatment. Despite its numerous clinical benefits, ICI therapy had induced various immune-related adverse events (irAEs) in different human organs. Male patients had a particularly higher risk of developing irAEs than their female counterparts (1–4). ICI combination therapies are correlated with higher grade irAEs with more serious implications and mortality as compared to ICI monotherapy (8). A study by Larkin et al. noted that 64–80% of patients who underwent therapy with CTLA-4 inhibitor, ipilimumab, developed irAEs, with 23% being Grade 3/4. Similarly, 79% of patients treated with a PD-1 inhibitor, pembrolizumab had irAEs, with 13% of those being Grade 3/4. When ipilimumab and nivolumab were combined, the incidence of irAEs reached 96% with 55% being Grade 3/4 (9).

Despite the higher incidence of irAEs, combination therapy's therapeutic effect still proves to be superior in terms of higher median progression-free survival and better objective response in patients. An objective response of 57.6% was achieved in combination therapy as compared to 40% in monotherapy users in advanced melanoma (9, 10). As such, combined ICI therapies do produce better clinical results but it also comes with a greater risk of irAEs for cancer patients.

Cardiovascular toxicity is among the high-grade irAE that should be given crucial attention due to its association with a high mortality rate (11). Major cardiovascular adverse events (MACE) include cardiac failure, myocarditis, pericarditis, vasculitis, etc., but MACE definition does vary across different journals. ICI-related myocarditis has an occurrence rate of about 1.14% compared to all other systemic irAEs. Although this prevalence rate may seem deceivingly low, the mortality rate is a gaping 25–50%. This calls for further analysis and as such, this review serves to provide a complete insight into the predisposing factors, mechanisms, diagnostic precision, and treatment plans for ICI-induced cardiotoxicity.

## The mechanisms of ICI-induced cardiotoxicity

Although increasing research interests have been focused on how ICI-induced cardiotoxicity evolves, the mechanism remains largely unknown. One prevalent theory is that PD-L1 is expressed in human cardiomyocytes, with its level enhanced by myocardial injury. Baban et al. (12) reported upregulation of PD-1 and PD-L1 upon ischemia/reperfusion after myocardial injury. Under normal situations, the binding of PD-L1 to PD-1 protects from the development of autoimmune myocarditis. Once PD-1 and PD-L1 inhibitors are used, the protective mechanism in cardiomyocytes is stripped, leading to cardiac tissue damage (13).

Cardiomyocytes and tumor cells have been found to exhibit similar lymphocytic environment properties and may thus share antigens with the T-cell receptors (14). Additionally, ICIs invigorate T-cell action by lifting the suppressive measures implemented on them by the tumor cells. It is therefore hypothesized that the cardiac cells and the cardiomyocytes expressing ICI-induced PD-L1 will face more active T-cell functions, which thus flare up as auto-immune reactions and finally manifest as cardiotoxicities. Consistent with this, Palaskas et al. and Michel et al. (8, 15) reported that homologous antigens present on tumor cells and cardiomyocytes are recognized by T-cell receptors exhibiting increased functioning capacity after administration of ICI. Animal experiments showed that mice with depleted PD-1 functions ended up with dilated cardiomyopathy with severe contraction difficulty, thus causing congestive heart failure. Diffused deposition of IgG was also spotted on cardiomyocytes in the mice which eventually died (16, 17).

CTLA-4 inhibitors have a similar mechanism of boosting T-cell response. The complete T cell activation requires T cell receptor (TCR) recognition, engagement by major histocompatibility complex (MHC)-bound (neo)antigens, and binding of co-stimulatory molecules like CD28 and B7 (CD80/CD86). CTLA-4 is an immune checkpoint molecule that negatively regulates T-cell activation by binding to B7 (CD80/CD86) molecules on antigen-presenting cells, opposing CD28-mediated co-stimulation (18). CTLA-4 inhibitors turn down the inhibitory roles of CTLA-4, leading to T-cell accumulation and increased T-cell activity in the cardiac environment (19) followed by fibroblast proliferation, neutrophil, and macrophage infiltration in cardiac tissues. Next, edema starts to develop and myocardial infarction is a common consequence (20). Results from animal experiments reveal that CTLA4 knockout mice developed lymphoproliferative disorder with excessive accumulation of activated T cells.

As such, in totality, it is safe to speculate that the over activity of T cells will cause an autoimmune response in ICI therapy patients. These theories of homologous antigen presentation demolished the premises of possible auto-antibodies that could have been formed against the cardiomyocytes (8).

## Types of cardiotoxicities

**Figure 1:**
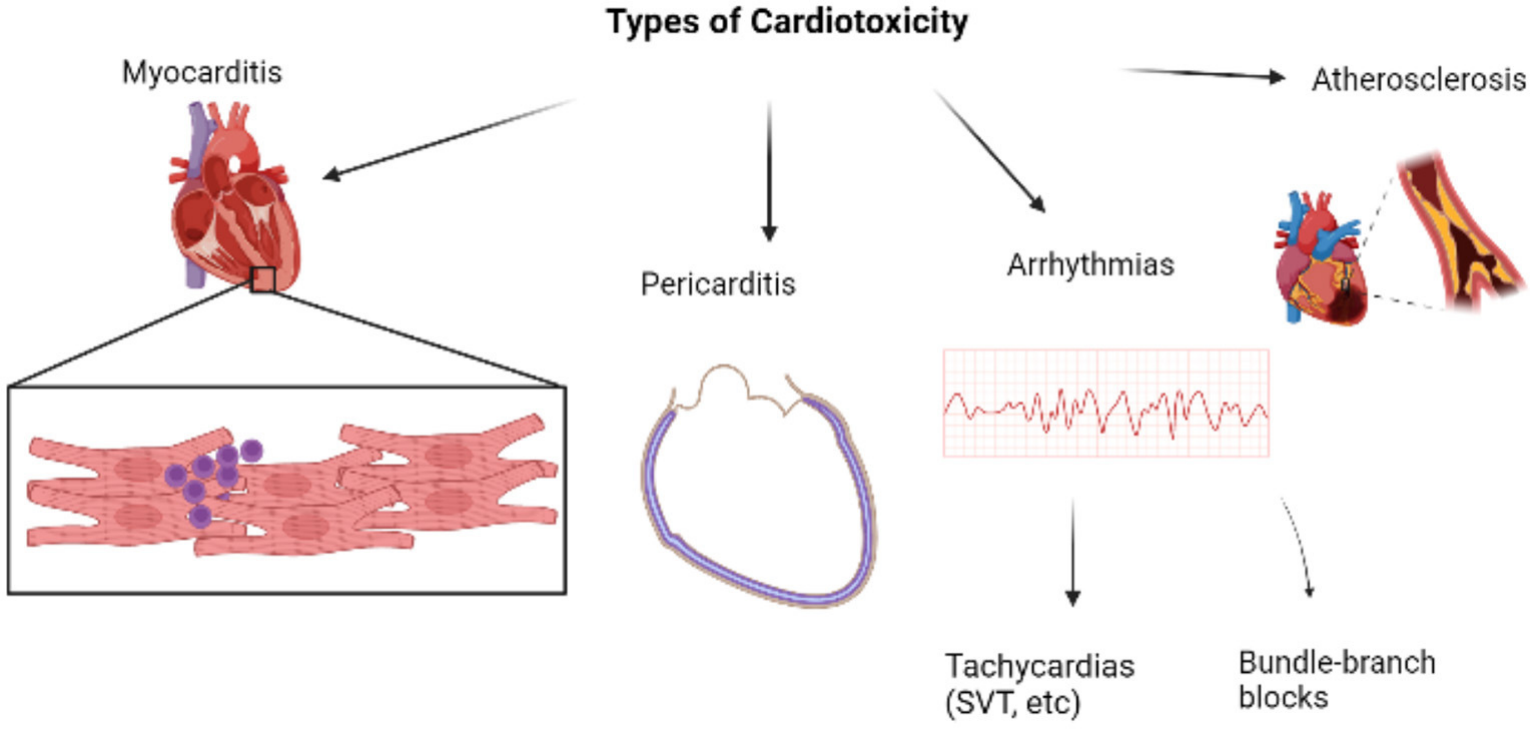
Overview of major types of cardiotoxicities [Created with BioRender.com].

Cardiotoxicity refers to the injury to myocytes which manifests as various cardiac conditions. When it comes to ICI-induced cardiotoxicities, certain irAEs are more common than others and vary in severity too (4). In worse cases, these cardiac conditions may even progress to a major cardiovascular event (MACE). Myocarditis stands first in the list of ICI-induced cardiotoxicity. Figure 1 is a brief overview of the types of cardiotoxicities that will be discussed in this review, with myocarditis being the focus owing to its highest prevalence amongst all cardiac irAEs.

Amongst all the systemic irAEs caused by ICIs, myocarditis has an occurrence rate between 0.27 and 1.14% in retrospective studies (5). Zooming into cardiac events, myocarditis accounted for 14.1% of cases, followed by pericarditis with 13.6% and conduction abnormalities in 6.86% of cases (5). Myocarditis has a histological picture depicted with CD3, CD4+, CD8+ lymphocytes and macrophages infiltrating the myocardium (21). The symptoms of myocarditis mimic that of acute heart failure and consist of chest pain, dyspnea, pulmonary edema, arrhythmias, elevated (N-terminal pro) brain natriuretic peptide (BNP/NT-proBNP), etc., (8, 14, 22). NT-proBNP was elevated in 66% of patients with diagnosed ICI-associated myocarditis, while Escudier et al. showed an elevated serum level of BNP/NT-proBNP in 100% of patients with ICI-related cardiotoxicity. An abnormal troponin level was observed in 94% of patients with ICI-associated myocarditis (23, 24). In the study by Zhang comprising 103 patients with ICI-associated myocarditis, 40% of patients developed major cardiovascular adverse events and 16.5% had a cardiovascular death over a follow-up time of 5 months. On the other hand, when compared to the 4.7 years of follow-up in 670 patients with myocarditis of other etiologies, 15% of patients experienced serious cardiovascular adverse events and 4% of patients died. The stark difference in the mortality rates of myocarditis triggered by ICI and other causes emphasizes the impact of ICI-induced myocarditis and the urgent need to address it immediately (25, 26). Myocarditis causes reported were mostly from the group that received combinatorial ICI therapy, particularly, recipients of a combination of CTLA-4 and PD-1 inhibitors (27, 28). As such, combination therapy seems to take a greater toll on the patients as compared to single-type ICI therapy.

Although myocarditis is the single most common adverse event, some patients' genetic makeup may confer protection against the development of myocarditis amidst ICI use. A study by Blyszczuk et al. (29) proved that the presence of the MyD-88 gene in patients enhanced the MyD-88/IL-1 axis function by promoting autoimmune-response of CD4+ cells. MyD-88 gene upregulates the genetic transcription of pro-inflammatory cytokines to flare inflammatory reactions and thus, plays a pivotal role in promoting fibrosis and progression toward myocarditis or heart failure (30). On the contrary, patients who were MyD-88^−/−^ had a diminished autoimmune response and thus were much less likely to develop myocarditis regardless of the triggers that patients were subjected to. As such, MyD-88^−/−^ patients have a reduced risk of developing myocarditis as compared to MyD-88^+/+^ individuals if started on ICI therapy. In this case, the genetic constitution can possibly provide some patients with a competitive edge (29, 31).

In a typical biopsy sample from a patient with myocarditis, T-cell– predominant lymphocytic infiltrate within the myocardium is commonly seen (8, 32). These T cell populations are clonally similar to those found in tumors, suggesting that cardiotoxicity is caused by antigen similarity; however, it may also be caused by reduced inhibition of self-reactive T cells (20).

Median time to the first clinical signs and suspicion of myocarditis usually occurs within an average of 34 days after the first ICI therapy, with about 81% of patients presenting within 3 months of starting therapy. As compared with non-MACE myocarditis cases, patients with MACE had a higher admission, peak, and discharge/final troponin T value (27).

Pericarditis is another common form of cardiotoxicity that manifests as chest pain, dyspnea, pericardial effusion, electrocardiogram (ECG) abnormalities, etc., in patients (14, 33). Pericarditis may also be reported with pericardial effusion, and even clinical tamponade. 15% of patients with cardiac abnormalities were diagnosed with pericardial diseases (13). Pericarditis could be caused by ICIs targeting an antigen that is homologous in both tumor cells and myocytes. Or it could be due to an anti-tumor response mounted against metastasis to the pericardium (34–36). Though it is not as fatal as myocarditis, pericarditis has a mortality rate of 13–21%. Pericardial patients frequently show ECG changes (37). Pericardial effusion or thickening may be identified on a CT scan while cardiac magnetic resonance (CMR) will show pericardial inflammation and/or fibrosis. Analysis of pericardial effusion fluid has displayed lymphocytes and plasma cells without evidence of malignant cells or microorganisms causing the symptoms (38). Pericarditis was more prominently associated with PD-1 and PD-L1 inhibitors as compared to CTLA-4 inhibitors (39, 40).

Other minor types of cardiotoxicities that are less prevalent include arrhythmias and vasculitis. Arrhythmias are conduction abnormalities that are spotted in various patients with ICI-induced cardiotoxicity and they may occur concurrently with myocarditis or pericarditis. Vasculitis, a term coined for the inflammation of blood vessels, is another aspect deserving of attention. It may affect blood vessels of various sizes and is typically classified into large, medium, and small-vessel vasculitis based on the size of the vessels involved. In a study, it was particularly observed that vasculitis in the form of temporal arteritis and polymyalgia rheumatica are way more common in ICI-induced pathology.

Takotsubo cardiomyopathy is another possible occurrence that has been reported. It is stress-induced cardiomyopathy that is quite rare in ICI-associated cardiotoxicity. Patients usually complain of having symptoms between 15 weeks and 8 months into treatment. It presents as transient cardiac regional wall motion abnormalities, new ECG changes, and elevated troponin and NT-proBNP (20). Clinicians may also see an apical ballooning pattern on echocardiograms (40).

Another concerning cardiotoxicity would be atherosclerosis. Although ICI usage has not been shown to induce any new atherosclerotic plaques, it has certainly been shown to aggravate inflammation of possible existing plaques and enhances the formation of necrotic debris. The elevated T cell response from ICI usage has characteristically increased the inflammatory response by promoting macrophage death, thus upregulating necrotic plaque formation (41). NLR family pyrin domain containing 3 (NLRP3) inflammasomes, which are part of the innate community of nucleotide-binding oligomerization domain-like receptors (NLR), are another triggering factor for atherosclerosis. These inflammasomes when activated, speed up the maturation of IL-18 and IL-1β, which are key cytokines in promoting atherosclerosis. Besides, the inflammasomes are also noted to enhance the migration of macrophages to stimulate foam cell formation and worsen endothelial dysfunction (42). A study by Quagliariello showed that patients who underwent ICI therapy trials had significantly elevated amounts of NLRP3 inflammasomes and Myd-88 which signal a pro-inflammatory side-effect of the ICIs. As such, it can be derived that the ICI does enhance the inflammatory role players in the immune system and as such, indirectly increase the chances of atherosclerosis but most importantly, the cytokine storm created will further multiply the chances of developing myocarditis (43).

### Predisposing factors for ICI-induced cardiotoxicity

Given the high mortality rate of ICI-induced cardiotoxicity, it is of clinical significance to determine predisposition factors both at individual and populational levels. Combination ICI therapy is one of the leading predisposing factors as supported by numerous sources (8). As opposed to monotherapy-induced cardiotoxicity, combinatory therapy is also notorious for more severe implications in patients (8, 15, 28). In a study involving 122 ICI-induced myocarditis patients, 65.6 and 44.4.% deaths event resulted from combination therapy and monotherapy, respectively (44). Next, some pre-existing medical conditions of the patients seem to put them at an increased risk of developing cardiotoxicities too. For instance, diabetes mellitus, obesity, pre-existing cardiac pathology or peripheral arterial disease, history of smoking, and dyslipidemia are some medical conditions that enhance the risk in patients (45).

Other risk factors include the use of cardiotoxic antineoplastic agents such as anthracyclines, anti-ErbB2 drugs, Raf and MEK inhibitors, VEGF tyrosine kinase inhibitors, underlying autoimmune diseases such as SLE, etc. As such, when diagnosing patients, the medical team needs to take the drug and medical history of patients into serious account (14).

### Tools for diagnosis of cardiotoxicity

It is crucial to make a timely and precise diagnosis for various adverse events related to ICI-induced cardiotoxicities. The time to perform the diagnostic tests should also be carefully considered as some tests could be used for screening purposes to distinguish suitable patients for ICI therapy while excluding the rest. Alternatively, screening tests could be done at regular intervals to identify any marked cardiac pathology.

One of the foremost diagnostic tools that are used at the forefront of diagnosis is biomarkers (46). Currently, there are no general recommendations for the usage of biomarkers in cardio-oncology patients but troponin levels are among one of the most routinely measured in cancer patients having cardiotoxicity. Cardiac biomarkers like troponin, brain natriuretic peptides (BNP), CK-MB, or total CK are signals for necrosis of myocytes and validate myocardial injury. Out of these, troponin and BNP take the helm in most biomarker studies. These markers are markedly elevated in cases of myocarditis. A final troponin T value of ≥1.5 ng/mL was associated with a significantly worse prognosis and a 4-fold increased risk of MACEs (15). In asymptomatic patients, cardiac troponin readings are the strategy most often recommended to screen for ICI-related myocarditis, despite this approach having inherent limitations (47). Increased troponin levels are indicative of myocardial injury but are not necessarily specific to myocarditis (48). As such, it is not wise to bank on a single value of troponin and a repeat troponin measurement should be done after 24 h of the first elevation. A steady increase in troponin levels can be expected in myocarditis cases (49). Natriuretic peptides are another vital group of biomarkers that are indicative of the stress-induced on the heart. But in its entirety, BNP should be evaluated with caution as they may elevate even from other inflammatory situations despite the patient having a normal filling pressure. Natriuretic peptides are not specific markers for myocarditis but are elevated in most cases of ICI-induced myocarditis (50).

ECG is another common form of diagnostic tool used to investigate any cardiac pathologies and it is a non-invasive technique with wide coverage (50, 51). In cases of myocarditis which is the most predominant form of ICI-induced cardiotoxicity, patients' ECGs may exhibit arrhythmia, ST-T wave abnormalities, PR segment changes, etc (50). For pericarditis, the ECG may exhibit typical changes in the presence of pericardial effusion, such as low QRS voltage, diffuse concave-upward ST-segment elevation, and tachycardia (52). Nonetheless, it should be noted that a significant number of myocarditis cases also produce a clinically normal ECG report (53).

Echocardiographs are also used in the diagnosis of the ventricular wall and segmental wall malfunctions, pericardial effusion, cardiac tamponade and the detection of anatomic defects (54). Echocardiograms can churn out measurements such as changes in LVEF, diastolic function, new wall motion abnormalities, or pericardial effusion (15). However, a study by Mahmood et al. (55) state that only about 49% of myocarditis patients present with abnormal echocardiography. Therefore, echocardiographs cannot be used as key diagnostic tools, but rather as a supporting examination. When echocardiographs show a decrease in myocardial deformation parameters, it is indicative of subclinical myocardial changes from cancer therapy. These parameter changes can be noted earlier than any changes in LVEF as seen in conventional 2D echocardiography. Early reduction in echocardiographic parameters is predictive of impending cardiotoxicity and speckle tracking echocardiography (STE) measuring global longitudinal strain (GLS) seems to be the most consistent parameter (56). GLS reflects the extent of myocardial edema and the exact regional localization detected in CMR (57). A decrease in GLS value early after chemotherapy is deemed to predict an impending decline in ejection fraction (EF) according to studies. The GLS was lower in patients with myocarditis presenting with both a reduced and preserved EF compared to controls during ICI therapy (57–60). As such, GLS can be used to identify patients at lower risk of subsequent adverse cardiac events and help avoid unnecessary immunosuppression (55). However, another echo measure, ejection fraction (EF) may be less useful for surveillance, because EF with myocarditis was normal in one-half of the cases. Having a normal EF did not prove much because 40% of patients with a normal EF still had a major cardiac event (61).

Cardiac magnetic resonance imaging (CMR) is the most preferred non-invasive method in the diagnosis of myocarditis and is considered superior to echocardiography. CMR reflecting myocardial edema and non-ischemic myocardial injury was highly indicative and more specific for myocarditis. CMR cannot be used alone but it sure can act as an adjuvant step in the diagnosis together with biomarkers, ECG, etc. The tissue characterization techniques such as gadolinium enhancement of a CMR can be of substantial aid in studying myocardial injury and hence, add credibility to the diagnosis (50, 62). The strengths of CMR lie in its excellent spatial resolution and remarkable tissue characterization. In cases of myocarditis, high signal intensity on T2-weighted images may reflect edema, myocardium may show greater contrast enhancement than skeletal muscle reflecting hyperemia and even scars may be noted (24). However, some papers have questioned the sensitivity of CMR in terms of myocarditis detection (63).

The golden standard test that holds the helm of the accurate myocarditis diagnosis is endomyocardial biopsy [EMB]. The accuracy of the histological details provided by a biopsy cannot be superseded by other diagnostic methods but the invasive nature of the biopsy serves as a major setback for this test. It also carries a cardiac perforation risk of <1% (24, 64).

**Figure 2:**
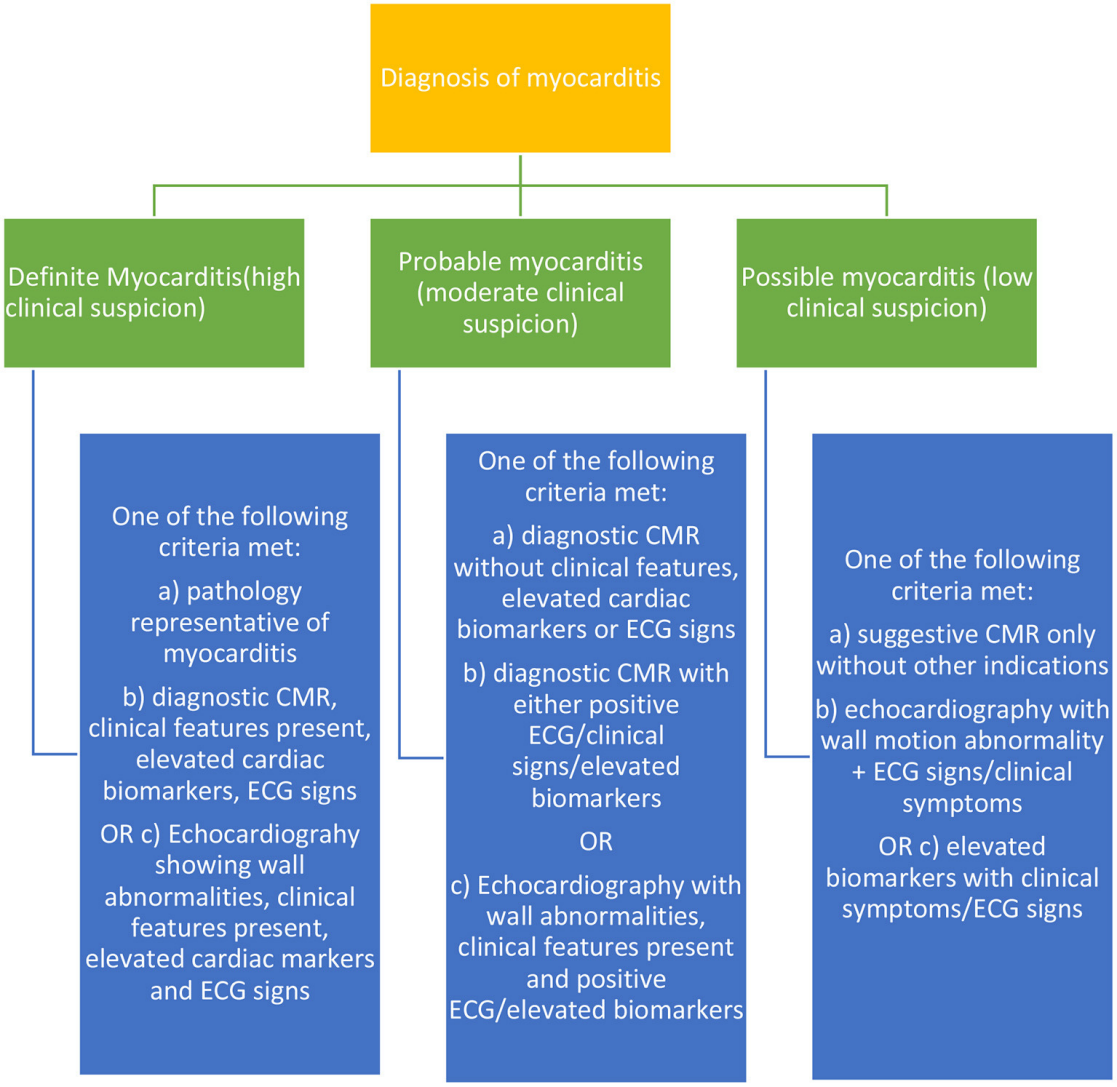
Diagnostic criteria for myocarditis, the most common ICI-induced cardiotoxicity.

In total, screening tests should be a necessity for patients undergoing ICI therapy, and these tests if decided to be conducted at regular intervals, should all be non-invasive. The EMB and other complementary tests which may be invasive should be pursued when there is a high suspicion of significant risk in patients in cases of myocarditis, etc based on other baseline tests. The following Figure 2 shows the categorical diagnostic criteria for myocarditis as described by many accredited journals.

### Treatment for ICI-induced cardiotoxicities

The success rate for treating ICI-induced cardiotoxicities lies in the timely diagnosis and appropriate treatment administration to patients. Various researchers have tried a multitude of ways to enhance the treatment plans for cardiotoxicities amidst ICI therapy. A consensus has been reached by most healthcare authorities about a suitable treatment plan, but no strict conformed treatment regimen has been laid out because the treatments are rather subjective to each patient. The treatment plan is customized to each patient and takes into account various factors such as the severity of the disease, comorbidities and pre-existing medical conditions in the patient, age, the possibility of relapse, etc (51).

Currently, there is no fixed protocol or guidelines designated by any association in terms of screening tests for the patients before starting ICI therapy although a study by Wang et al. (65) proposed cardiac troponin measurements at regular intervals before and during ICI therapy. This article suggested that measurements can be spaced from 2 weeks before ICI treatment to 1 and 3 months after starting treatment. Another article by Puzanov et al. (66) stated that one measurement can be taken before the start of treatment and every week after starting treatment for the first 6 weeks.

**Figure 3:**
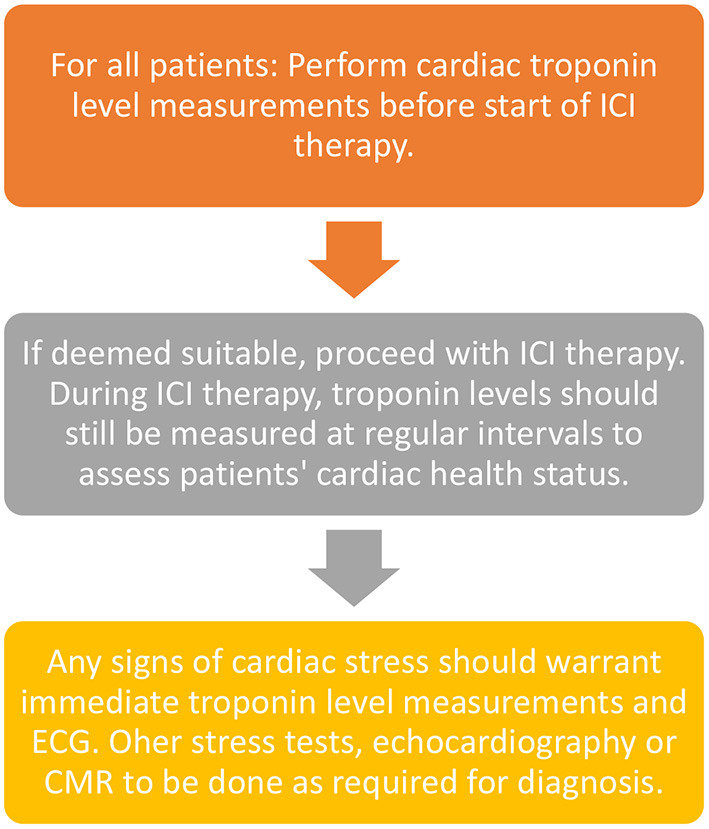
Monitoring ICI patients for cardiotoxicity.

Although cardiac troponin levels and other biomarkers form the mainstream first-line monitoring techniques, some patients with pre-existing cardiac conditions or other chronic diseases may warrant additional examinations such as ECG, stress tests or even CMRs spaced apart although the latter is done rarely as part of the monitoring regime. A summary of preferred monitoring techniques is given in Figure 3 below.

When it comes to ICI-induced myocarditis, the treatment plan is to immediately stop ICI therapy and start the patient on a high dose of corticosteroids (1–2 mg/kg/day) to suppress the immune system until the cardiac function is restored. For other kinds of organ system irAEs, ICI therapy may still be potentially continued with appropriate treatment but in the case of myocarditis, it is essential to stop the ICI therapy with immediate effect given the high mortality involved in myocarditis (40, 67–69). Corticosteroid treatment should be administered in time otherwise if the disease progresses, it may lead to fulminant myocarditis in patients (67, 70, 71).

High-dose corticosteroids (>500 mg/day) administered promptly within 24 h of the onset of cardiac symptoms provided the most satisfactory patient improvement. A study by Zhang et al. divided methylprednisolone doses into low (<60 mg/day), moderate (60–500 mg/day), and high (501–1,000 mg/day). These three doses were administered on the 1st day to different sets of ICI-induced cardiotoxicity patients and continued as part of the treatment plan (72). Data proved that immediately starting patients on a high dose of corticosteroids reduced the risk of patients developing MACEs and paved the way for better recovery of left ventricular function (27) as compared to the lower doses. The time of the first administration of corticosteroids also seems to have a profound impact on creating better outcomes for patients. In a trial, the corticosteroids were administered within 24 h, between 24 and 72 h, and after 72 h from the onset of cardiac symptoms in three different groups of patients, respectively. Patients given corticosteroids within 24 h faced lesser elevation of troponin at discharge as compared to the other two groups. Regardless of the dosage, patients administered corticosteroids within 24 h of symptom onset faced better treatment outcomes.

Similar corticosteroid treatment plans were recommended for the other cardiotoxicity types together with supportive treatment like a pericardial drain for pericardial effusion, antibiotics for sepsis, etc. For hemodynamically stable patients with acute myocarditis or pericardial effusion, an initial dose of 1 mg/kg/day of intravenous methylprednisolone may be sufficient in the acute setting, followed by a slow oral prednisone taper over a month or longer (49). If corticosteroids fail to produce the desired effect, other drugs such as mycophenolate mofetil, methotrexate, IVIG, plasmapheresis, anti-thymocyte globulin, rituximab, infliximab, etc., can be employed (67, 73).

**Figure 4:**
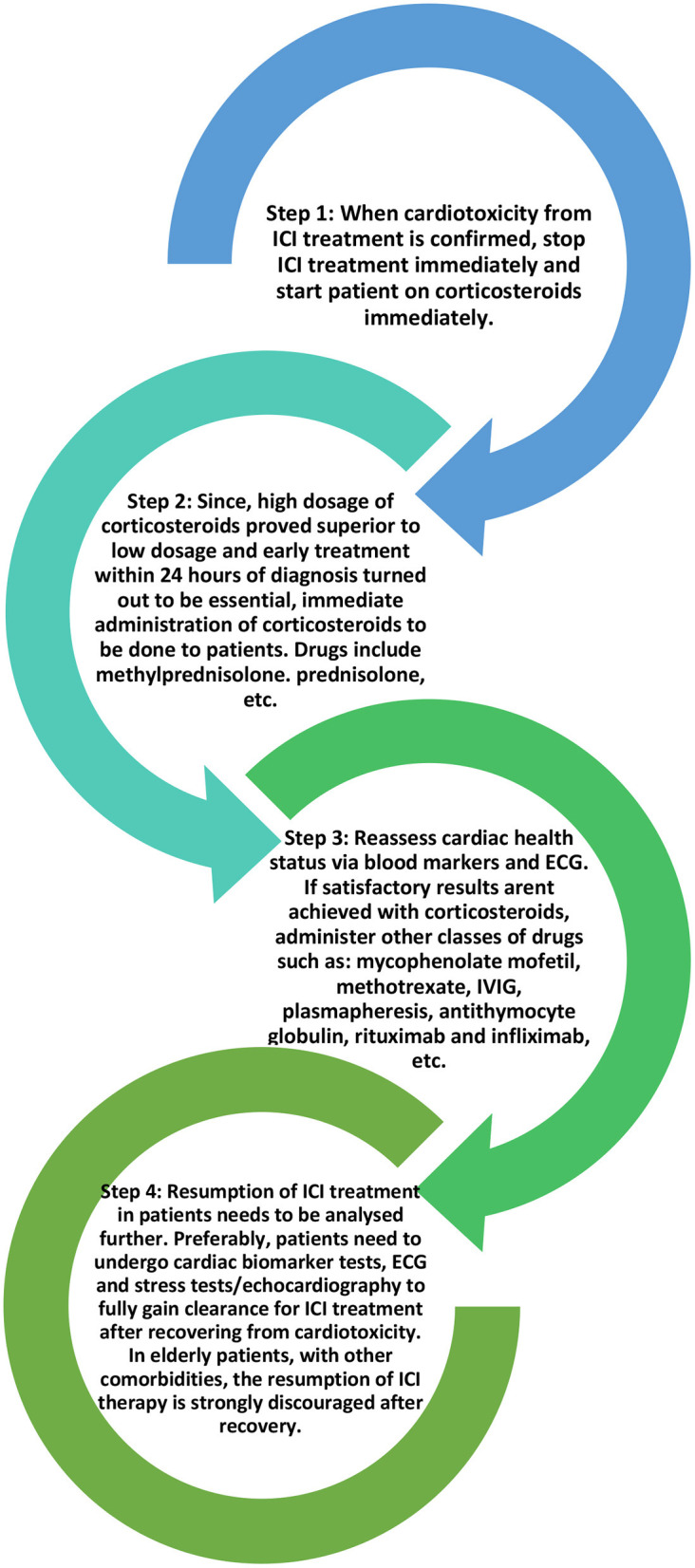
General treatment protocol for ICI-induced cardiotoxicity.

Recent studies have suggested that the usage of CTLA-4 agonists such as abatacept or belatacept could tone down the co-stimulation of T-cells in the PD-L1/PD-1 pathways, as such preventing the autoimmune action of T-cells on cardiac myocytes (74, 75). Other spectra of studies have suggested the usage of IL-1β blockers such as canakinumab to reduce the severity of cardiotoxicity. These drugs supposedly decrease the mortality rate and improve cardio-pulmonary functions (76). However, these studies should be subjected to further scrutiny before addition to the usual drug regimen. Figure 4 below shows the current general treatment protocol for ICI-induced cardiotoxicity.

Despite these pharmaceutical treatments making a breakthrough in treating ICI cardiotoxicity, it is worthwhile to explore non-pharmaceutical approaches as well. The usage of nutraceuticals like supplements, ascorbic acid, etc., may potentially reduce the impact and risks of developing ICI toxicity too. A study by Berretta et al. analyzed the various effects of ascorbic acid in treating cardiovascular disease patients and documented that ascorbic acid plays a role in enhancing endothelial function and reducing blood pressure. Ascorbic acid seems to annul the oxidizing effect of iron and oxygen on cells, thus reducing the chances of radical injury to myocytes. It is also believed to keep lipid peroxidation at bay to ensure no cardiotoxicity (77, 78). Another aspect for consideration would be the usage of curcumin and its analogs to enhance the ICI treatment potential. The usage of PD-1 inhibitors has been shown to upregulate the proliferation of PD-1^+^ T regulatory (Treg) cells in patients who concurrently receive radiation therapy. This reduces the efficacy of ICI therapy in some individuals. Curcumin analogs such as GO-Y030 suppress the proliferation of Treg cells and induce the production of ROS in radicals in them, thus promoting ICI treatment efficacy (79, 80). With the suppressed activity of Treg cells, the concurrent use of these analogs and the stimulated T cell immune response from ICI therapy produces an additive effect to combat tumor cells.

Table 2 sums up the content of some of the latest research papers on cardiotoxicity and highlights key issues discussed in this review (types of cardiotoxicity, methods of diagnosis, treatments, etc). It serves as a comparison of data in different trial settings.

**Table 2:** Summary of latest research reviews on ICI-induced cardiotoxicity.

**Research review**	**Types of immune checkpoint inhibitors used**	**The most prevalent cardiotoxicity encountered**	**Prevalence of various cardiotoxicities**	**Tools for diagnosis**	**Treatment methods administered**
Salem et al. (5)	Combination therapy (CTLA-4 and PD-1 inhibitors) vs. monotherapy (PD-1)	Myocarditis	Myocarditis (*n =* 122)/ (0.39%), Pericarditis (*n =* 95) / (0.3%), Vasculitis, etc (*n* = 82) / (0.26%) prevalence is relative to all irAEs.	ECG, cardiac biomarkers, echocardiography	High-dose glucocorticoids, abatacept
Escudier M et al. (23)	Combination therapy (CTLA-4 and PD-1 inhibitors), monotherapy (PD-1/PD-L1)	Atrial fibrillation	Atrial fibrillation (30%), ventricular arrhythmias (27%), and conduction disorders (17%) prevalence relative to cardiovascular irAEs	ECG, cardiac biomarkers, TTE	Corticosteroid therapy
Michel et al. (8)	Combination therapy (CTLA-4 and PD-1 inhibitors), monotherapy (PD-1/PD-L1)	Myocarditis	Myocarditis (most prevalent out of all cardiotoxicities), pericarditis, Takotsubo syndrome, acute coronary syndrome.	ECG, cardiac biomarkers, echocardiography, CMR	Glucocorticoids, mycophenolate, mofetil, infliximab, anti-thymocyte globulin, etc.
Slawinski et al. (24)	Combination therapy (CTLA-4 and PD-1 inhibitors), monotherapy (PD-1/PD-L1)	Myocarditis	Myocarditis (14.1%), pericarditis (13.6%), conduction abnormalities (6.86%)	Cardiac biomarkers, ECG, echocardiography, CMR	High dose corticosteroids, immunoglobulin, plasmapheresis, mycophenolate mofetil
Shalata et al. (40)	Combination therapy (CTLA-4 and PD-1/PD-L1 inhibitors), monotherapy (PD-1/PD-L1)	Myocarditis	Myocarditis, pericarditis, takotsubo syndrome, conduction abnormalities	Cardiac biomarkers, ECG, CMR	High-dose corticosteroids, mycophenolate mofetil, infliximab or anti-thymocyte globulin, and other supportive treatment
Spallarossa et al. (68)	ICI monotherapy	Myocarditis	Myocarditis, pericarditis, takotsubo syndrome, acute coronary syndrome, and vasculitis	Cardiac biomarkers, ECG, Chest X-ray,	High-dose corticosteroids, mycophenolate mofetil, infliximab, or anti-thymocyte globulin
Esposito et al. (57)	Combination therapy (CTLA-4 and PD-1/PD-L1 inhibitors), monotherapy (PD-1/PD-L1)	Myocarditis	Myocarditis (0.39%), pericardial diseases (0.30%), Myocardial infarction (0.53%), supraventricular arrhythmias (0.71%) prevalence relative to all irAEs. Since only the most severe cases of myocarditis were reported, in reality, the cases of myocarditis may be more than the reported tally.	Cardiac biomarkers, ECG, echocardiography, CMR	Corticosteroids, IVIG, plasmapheresis, mycophenolate mofetil, infliximab, tacrolimus, abatacept, etc.
Kazama et al. (81)	Monotherapy with CTLA-4, PD-1, and PD-L1 inhibitors	Arrhythmias	Arrhythmias (3.6%), angina pectoris (2.2%), pericardial effusion (1.4%), myocarditis (0.7%), vasculitis (0.7%) prevalence relative to all irAEs that occurred in 138 patients involved in the study	-	-
Shindo et al. (82)	Monotherapy (PD-1 and PD-L1 inhibitors) and combination therapy (PD-1 and CTLA-4 inhibitors)	Myocarditis	Myocarditis, (0.06-0.27%), myositis, etc. prevalence of myocarditis with respect to all irAEs	Cardiac biomarkers, ECG, echocardiography, cardiac MRI	High dose corticosteroids and discontinuation of ICI therapy

## Future work

The given predictive biomarkers that help to identify patients and organ systems at risk well-before ICI therapy are still not well-defined (20). Patients with comorbidities are expected to face more risk of developing cardiotoxicity. Herein, cardiotoxicity may be seen in the most unexpected of patients for its unpredictability.

The recent COVID-19 infections have taken a toll on patients' immune response as well by triggering CD4+ T cell immune response and activating a series of pro-inflammatory cytokines, causing notable tissue damage in various organs (83). There has been a hesitance to employ immune regulatory treatment in cancer patients with simultaneous COVID-19 infections for fear of potentiating viral replication and reducing host immune viral clearance (84). Besides, the pro-inflammatory response from coronavirus infection may further magnify the effects of ICI therapy. Covid-19 viral infection was further noted to enhance the chances of patients with comorbidities developing myocarditis (85). As such, covid-19 patients who are also undergoing ICI therapy should be subjected to further monitoring.

Rechallenging patients with ICI therapy after resolving cardiotoxicity is also a crucial consideration. Dolladille, C et al. have recently published that irAEs associated with high mortality rates are less likely to cause recurrence upon re-challenge with ICI. As such, since myocarditis poses a high mortality risk, it may be likely that the chances of recurrence are lower with rechallenge and the patients who have recovered successfully from ICI-induced myocarditis can be subjected to ICI again (86). However, this matter is yet to be further disputed.

The addition of non-pharmacologic interventions to the ICI therapy may annul some effects and thus provide an overall balanced immune response in patients. However, since ICI therapy is still an evolving field, the interaction between nutraceuticals and ICI drugs should be further explored via trials before experimenting on various cancer populations. Although the nutraceuticals may counter the downside effects of ICIs, the effect and overall balance may vary from one individual to another. Thus, no concrete conclusion about the usage or addition of nutraceuticals to the current pharmacological intervention be made. This is an aspect that needs to be further explored as well.

## Author contributions

All authors listed have made a substantial, direct, and intellectual contribution to the work and approved it for publication.

## Funding

This work was supported by the National Natural Science Foundation of China (Grant No. 81970331).

## Conflict of interest

The authors declare that the research was conducted in the absence of any commercial or financial relationships that could be construed as a potential conflict of interest.

## Publisher's note

All claims expressed in this article are solely those of the authors and do not necessarily represent those of their affiliated organizations, or those of the publisher, the editors and the reviewers. Any product that may be evaluated in this article, or claim that may be made by its manufacturer, is not guaranteed or endorsed by the publisher.
